# Discrimination and Harassment Experiences of Autistic College Students and Their Neurotypical Peers: Risk and Protective Factors

**DOI:** 10.1007/s10803-022-05729-2

**Published:** 2022-09-14

**Authors:** Sohyun An Kim, Lauren Baczewski, Maria Pizzano, Connie Kasari, Alexandra Sturm

**Affiliations:** 1 Center for Dyslexia, Diverse Learners, and Social Justice, University of California Los Angeles, 3005B Moore Hall, Los Angeles, CA 90095-1521 USA; 2 Charter College of Education, California State University Los Angeles, Los Angeles, USA; 3 Department of Education, University of California Los Angeles, Los Angeles, USA; 4 Semel Institute for Neuroscience, University of California Los Angeles, Los Angeles, USA; 5 Center for Autism Research and Treatment, University of California Los Angeles, Los Angeles, USA; 6 Department of Psychological Science, Loyola Marymount University, Los Angeles, USA

**Keywords:** Autism spectrum disorder, Postsecondary education, Discrimination, Harassment, Faculty support, Habits of mind, Neurodiversity

## Abstract

This study examines autistic and non-autistic college students’ experiences of discrimination and harassment and identifies protective and risk factors. A nationwide survey was used to match autistic students (N = 290) and non-autistic students (N = 290) on co-occurring diagnoses and demographic characteristics. Multiple regression and interaction analysis revealed that faculty support was protective against discrimination and harassment regardless of autism status. Habits of mind was particularly protective for autistic students against harassment. Any student who engaged in school-facilitated events was more likely to experience discrimination and harassment, but the risk was heightened for autistic students. Findings highlight the importance of faculty support in fostering positive interpersonal experiences on campus, and demonstrate the need to address deeper college campus issues with respect to neurodiversity.

## Introduction

Nearly half (43.9%) of transition-age autistic adults further their education by enrolling in postsecondary schools such as four-year universities, community colleges, and vocational schools (Newman et al., 2011). Emerging evidence suggests that autistic college students encounter high rates of discrimination and harassment, which may impact their college experience and retention (Anderson et al., 2019; Gelbar et al., 2015; Jackson et al., 2018). Discrimination and harassment may encompass a wide range of negative interpersonal experiences. In the current study, discrimination refers to negative experiences related to verbal or cyber bullying, deliberate social exclusion, receiving offensive phone calls, and/or exposure to offensive images or items. The term harassment describes more severe forms of victimization such as being threatened with or experiencing physical or sexual assault, and/or damaged personal property (CIRP Factor Technical Report, 2019).

### Discrimination and Harassment

Social rejection is one form of discrimination that autistic students report experiencing in college, both from peers and faculty (Gelbar et al., 2015; Jackson et al., 2018). Autistic college students report higher rates of verbal and physical bullying, and deliberate social exclusion when compared to their NT peers. Additionally, they report being the victims of lies and rumors more frequently than both their NT peers and their peers who report other disabilities (McLeod et al., 2019). Autistic college students are repeatedly found to be more likely than their NT peers to feel lonely and different from others, with a higher incidence of mental health challenges (Anderson et al., 2019; Gelbar et al., 2015; Jackson et al., 2018; McLeod et al., 2019; White et al., 2011).

Evidence of the negative effects of peer victimization on autistic adolescents suggests that verbal and physical victimization by peers is associated with negative social, emotional, and academic outcomes for students (i.e., mental health, self-esteem, school sense of belonging, and academic achievement) (Adams et al., 2016; Ashburner et al., 2019; Chou et al., 2020; van Schalkwyk et al., 2017). Despite the likely impact of discrimination and harassment on student experiences and wellbeing in autistic young adults, very few studies to date have examined the discrimination and harassment experiences reported by autistic college students in comparison to their non-autistic peers. Given that autistic students report higher rates of mental health challenges compared to neurotypical peers (e.g., Jackson et al., 2018), the current study aimed to examine autistic students’ experiences of discrimination and harassment, and to investigate potential buffers against these experiences.

### Predictors of Discrimination and Harassment

Several factors impact students’ college experiences and mental health, including both student- level characteristics and institutional-level factors (Byrd & McKinney, 2012). Student characteristics include those traits and behaviors that the student holds upon entering the college context, while institutional factors are aspects of the college environment that the student interacts with (Astin, 1993; Kitzrow, 2003). Several student and institutional factors contribute to and buffer against negative interpersonal experiences on college campuses (Cabrera et al., 1999), including students’ identities, habits of mind, faculty support, and students’ social integration within their campus community.

#### Student-Level Factors

Among the general college student population, identifying as a member of a minoritized group (e.g., racial/ethnic and/or sexuality and gender minorities) is a student-level characteristic associated with increased discrimination experiences (Stevens et al., 2018). Racial/ethnic minority college students experience significantly more discrimination than their White counterparts, with negative impacts on academic success and mental health (Stevens et al., 2018). LGBTQ + students face similar negative outcomes as a result of victimization experiences, as discrimination is related to greater depressive symptoms and a greater likelihood to attempt suicide (Busby et al., 2020). Recent decades have seen increased recognition and understanding of disability, and autism specifically, as its own identity group, whose members face similar stressors to those in other minoritized groups (Botha & Frost, 2020). Consequently, autistic students who are at the intersection of multiple minoritized identities can experience a heightened level of discrimination experiences (Hillier et al., 2020). Despite this fact, few studies to date have examined whether autistic students report experiencing more discrimination and harassment in college compared to non-autistic peers, and whether certain factors may buffer against discrimination and harassment.

In addition to identity, students’ habits of mind, defined as ways of approaching learning that are both intellectual and practical (Johnson, 2013), can have positive impacts for autistic students’ college experiences. College readiness can be defined in terms of intellectual behaviors and educational experiences that encompass habits of mind and rhetorical skills (Johnson, 2013). Costa & Kallick (2008) conceptualized habits of mind as a disposition towards behaving intelligently when confronted with problems, or behavioral habits associated with effective learning. Some of these behaviors involve problem-solving, taking intellectual risks, analyzing multiple sources of information to draw conclusions, and accepting mistakes as part of the learning process. These behaviors can empower students to effectively solve problems in their academic and social situations (Burgess, 2012). While fostering habits of mind is often linked to positive academic outcomes (Costa & Kallick, 2008), it is also linked to increased interpersonal and social skills such as listening with understanding and empathy, persistence, applying past knowledge to new situations, and managing impulsivity (Burgess, 2012). Habits of mind were also linked to decreased challenging behaviors in students with social and emotional difficulties or developmental disabilities (Burgess, 2012). While less is known about how habits of mind are related to college students’ negative interpersonal experiences, it is worthwhile to investigate if these skills are important in fostering positive college experiences for minoritized groups such as autistic students.

Moreover, social and academic integration are critical to a successful college experience, in particular for disabled students (Tinto & Russo, 1994; Vaccaro et al., 2015). A student’s ability to socially integrate with their campus community and feel a sense of belonging depends upon several factors, including the inclusivity of the campus environment, the receptivity of the student body, and exposure to social opportunities (Hurtado et al., 2015). Importantly, feeling included within the student body has been shown to mitigate or buffer against the effects of discrimination and bias among ethnic minority students (Hurtado et al., 2015). It is therefore also important to examine whether greater engagement with one’s institution may buffer against discrimination and harassment experiences for autistic students.

#### Institutional-Level Factor

Beyond student-level characteristics, institutional-level factors play an instrumental role in student experience (Byrd & McKinney, 2012; Nora et al., 1996).

Validating and supportive experiences with faculty and staff predict greater student academic integration and sense of belonging, which mitigates the effects of discrimination among minority students (Hurtado et al., 2015). Thus, faculty support is another institution-level factor that may buffer against the negative impact of discrimination and harassment for minoritized students, and it is critical to explore whether this is the case for autistic college students.

Overall, studies have yet to examine student and institutional-level factors related to discrimination and harassment experiences among autistic students in college. To support the wellbeing and success of autistic students in the higher education environment, we must understand whether they experience discrimination and harassment at higher rates than their non-autistic peers. Furthermore, it is critical that we explore and identify factors that may buffer against the negative experiences such as discrimination and harassment. Identifying these risk and protective factors may facilitate a greater understanding of the autistic student experience and inform higher education programming focused on more inclusive campus environments. This is the first study, to our knowledge, that examines the discrimination and harassment experiences of autistic students in comparison to non-autistic peers and explores factors that may buffer against the negative effects of those experiences. Using a sample of 290 self-identified autistic college students and non-autistic peers matched on co-occurring diagnoses and demographic characteristics, the present study investigates the following research questions: (1) Do autistic college students report significantly more instances of discrimination and harassment on campus compared to their non-autistic peers? and (2) What are the protective factors for discrimination and harassment on campus at the student-level and institutional-level as reported by autistic and non-autistic college students?

We hypothesized that autistic college students may report significantly more discrimination and harassment on campus compared to their non-autistic peers. We further hypothesized that students’ habits of mind and involvement in campus-facilitated activities may serve as student-level protective factors, and faculty support may serve as institutional-level protective factor for experiencing discrimination or harassment on campus.

## Methods

### Participants

The present study used data from the Diverse Learning Environment (DLE) national survey developed by the Cooperative Institutional Research Program (CIRP) at the Higher Education Research Institute (HERI) at the University of California Los Angeles (UCLA) (HERI, 2022). The DLE is annually administered nationwide by individual 2-year and 4-year colleges to students who have obtained at least 24 credit hours in 2-year colleges and 2nd and 3rd year students at 4-year colleges. The DLE captures student perceptions of faculty, staff, and peers and also asks students to report on their school’s climate relating to diversity and student outcomes (HERI, 2022). Beginning in 2017 and in every subsequent year, students were asked to select all that apply to the following question: “Do you have any of the following disabilities or medical conditions?” Response options included *learning disability (e.g., dyslexia)*, *attention-deficit/hyperactivity disorder (ADHD)*, *autism spectrum disorder*, *physical or sensory disability (e.g., speech, sight, mobility, hearing, *etc*.)*, *chronic illness (e.g., cancer, diabetes, autoimmune disorder, *etc*.)*, *psychological disorder (e.g., depression, anxiety, PTSD, *etc*.)*, and *other*. Students were included in the present study if they: (1) self-reported a diagnosis of autism spectrum disorder (ASD) or (2) reported not to have a diagnosis of autism. Students in the sample completed the survey at one time during the survey years 2017–2020.

A total of 53,116 college students responded to the DLE survey in the years 2017 to 2020. Of those, 474 students reported a diagnosis of autism spectrum disorder (ASD). Case–control matching was conducted to match an autistic sample to the non-autistic sample based on their reported race (White as reference group), gender, family income range, institution type, first-generation college student status, co-occurring diagnoses (i.e., ADHD, learning disabilities, psychological disorders and physical disabilities) using the case control procedure in SPSS version 27. The matched samples included 290 autistic students and 290 non-autistic students, with a total of 580 students in the sample. Of the 474 autistic students in the entire dataset, 184 cases were not matched and therefore were excluded from the sample during the case–control matching procedure. Mean comparisons of predictor variables, outcome variables, and demographic characteristics between the 290 matched and 184 unmatched (i.e., excluded) autistic students are summarized in Tables 1 and 2. Lastly, while the dataset used for the current study included multiple cohorts of student surveys from 2017 to 2020, it was confirmed that no cases were counted more than once in the current sample.

**Table 1:** Mean comparisons of factor scores between matched and unmatched autistic students

	Matched ASD cases (N = 290)	Unmatched ASD cases (N = 185)	p
Discrimination	5.82	6.19	> 0.05
Harassment	4.02	4.30	> 0.05
School involvement	3.99	4.11	> 0.05
Faculty support	12.10	11.84	> 0.05
Habits of mind	16.64	16.48	> 0.05

**Table 2:** Demographic information of matched and unmatched autistic students

Student characteristic N (%)	Matched ASD cases (N = 290)	Unmatched ASD cases (N = 185)	p
Race			
White	184 (63.4%)	117 (63.6%)	> 0.05
Two or more races/non-Hispanic	41 (14.1%)	29 (14.1%)	
Hispanic—any race	19 (6.6%)	15 (8.2%)	
Asian/American Indian/Hawaiian	31 (10.7%)	10(5.4%)	
Black/non-Hispanic	9 (3.1%)	12 (6.5%)	
Unknown	6 (2.1%)	4 (2.2%)	
First generation college students	33 (11.4%)	17 (19.8%)	0.044
Gender identity			
Male	136 (46.9%)	97 (54.5%)	0.003
Female	127 (43.8%)	52 (29.2%)	
Other	27 (9.3%)	29 (16.3%)	
Annual family income			
under $30,000	98 (33.8%)	43 (23.2%)	> 0.05
$30,000–$50,000	34 (11.7%)	20 (10.8%)	
$50,000–$100,000	77 (26.6%)	46 (24.9%)	
$100,000–$200,000	65 (22.4%)	38 (20.5%)	
$200,000 or more	16 (5.5%)	13 (7.0%)	
Institution type			
University	50 (17.2%)	74(40.2%)	< 0.001
4 Year College	180 (62.1%)	84 (45.7%)	
2 Year College	60 (20.7%)	26 (14.1%)	

### Measures

#### Demographic Characteristics

Demographic characteristics of the sample included: (1) Race/ethnicity, (2) gender (male, female, other), (3) family income range (under $30,000, $30,000 to $50,000, $50,000 to $100,000, $100,000 to $200,000, $200,000 or more), (4) institution type (2-year college, 4-year college, 4-year university), and (5) first-generation college student status (yes, no).

#### Discrimination

To measure discrimination, a latent variable indicated by five items from the DLE survey items that represent the maximum likelihood score of students’ experiences with subtle forms of discrimination was created. Students were asked, “Please indicate how often you have personally experienced the following forms of bias/harassment/discrimination at this college” and rated the frequency of occurrence of each of the following: verbal comments, cyberbullying (e.g., emails, texts, social media), exclusion (e.g., from gatherings, events), anonymous phone calls, and offensive visual images or items. Response options included: 1—“Never,” 2—“Seldom,” 3—“Sometimes,” 4—“Often,” and 5—“Very often.” Due to the violation of multivariate normality, the “MLF” estimator (maximum likelihood estimation with standard errors based on the first-order derivatives) was used. In addition, the variables included in this model passed the Missing-Completely-at-Random (MCAR) test (χ^2^ = 7.629, df = 4, p = 0.106) (Little, 1988), and therefore full-information maximum likelihood (FIML) was used to handle missing data. Consistent with established model fit standards, model satisfying the following criteria were considered to have adequate model fit and were included in the present study (a) RMSEA ≤ 0.06, (b) SRMR ≤ 0.08. and (c) CFI ≥ 0.95 (Hu & Bentler, 1999). The unidimensional confirmatory model evidenced adequate fit (χ^2^ = 18.346, *p* = 0.003, df = 5, CFI = 0.959, RMSEA = 0.068, SRMR = 0.035), and Cronbach’s alpha also indicated adequate internal consistency of the items (α = 0.83). Next, discrimination factor scores based on this confirmatory model were calculated for each student in the sample.

#### Harassment

To measure harassment, a latent variable indicated by four items from the DLE survey items that assess the frequency that students experience more severe forms of threats or harassment was created. Students were asked, “Please indicate how often you have personally experienced the following forms of bias/harassment/discrimination at this college” and rated the frequency of occurrence of physical assaults or injuries, threats of physical violence, damage to personal property, been sexually harassed. Response options included: 1—“Never,” 2—“Seldom,” 3—“Sometimes,” 4—“Often,” and 5—“Very often.” Due to the violation of multivariate normality, the “MLM” estimator (maximum likelihood) was used. In addition, the variables included in the model did not pass the MCAR test (χ^2^ = 26.819, df = 15, p = 0.03) (Little, 1988), and therefore listwise deletion was used to handle missing data as opposed to FIML. Five hundred and seventy four out of the 580 cases in the sample were used. The unidimensional confirmatory model evidenced good fit (χ^2^ = 0.583, *p* = 0.747, df = 6, CFI = 0.999, RMSEA = 0.020, SRMR = 0.010), and Cronbach’s alpha also indicated adequate internal consistency of the items (α = 0.85). Harassment factor scores based on this confirmatory model were then calculated for each student in the sample.

#### Protective and Risk Factors of Discrimination and Harassment

To identify predictors of discrimination and harassment, three latent variables were created in separate measurement models. The three latent variables were *school involvement*, *faculty support*, and *habits of mind*. Lavaan package (Rosseel, 2012) in R was used to estimate each latent variable, where the factor loading for the first survey item representing the latent variable was fixed to 1 and all other factor loadings were estimated. Due to the ordinal nature of the survey items, diagonally weighted least squares (DWLS) was used to estimate the model parameters. Additionally, the full weight matrix was used to compute robust standard errors and mean-and variance adjusted test statistics.

##### School Involvement

School involvement was indicated by five items and evidenced good fit (χ^2^ = 291.810, p < 0.01, df = 10, CFI = 0.978, RMSEA = 0.046, SRMR = 0.071). Cronbach’s alpha also indicated adequate internal consistency of the items (α = 0.90). A 6th item assessing participation in intercollegiate athletics (“Since entering this college, have you: joined a social fraternity or sorority, participated in leadership training, joined an ethnic or culturally based fraternity or sorority, participated in a student-run political club, joined a racial/ethnic student organization, and played intercollegiate athletics,?”) was excluded from the school involvement factor due to low internal consistency of the items (α = 0.55). The five variables included in the model passed the MCAR test (χ^2^ = 34.601, df = 29, p = 0.218) (Little, 1988), and therefore FIML was used to handle missing data. Lastly, *school involvement* factor scores based on this confirmatory model were calculated for each student in the sample.

##### Faculty Support

Faculty support was indicated by five items and measured students’ perceived support and encouragement from their faculty. Students were asked, “Please indicate the extent to which you agree or disagree with the following statements” and rated frequency of each of the following: *faculty encouraged me to ask questions and participate in discussion*, *faculty empower me to learn here*, *faculty believe in my potential to succeed academically*, *at least one staff member has taken an interest in my development*, and *at least one faculty member has taken an interest in my development*. Answer options included: 1—“Strongly disagree,” 2—“Disagree,” 3—“Agree,” and 4—“Strongly agree” for each item. The variables included in the model passed the MCAR test (χ^2^ = 21.160, df = 17, p = 0.219) (Little, 1988), and therefore FIML was used to handle missing data. The unidimensional confirmatory model evidenced adequate fit (χ^2^ = 18.427, p = 0.001, df = 4, CFI = 0.989, RMSEA = 0.079, SRMR = 0.020), and Cronbach’s alpha also indicated adequate internal consistency of the items (α = 0.83). *Faculty Support* factor scores based on this confirmatory model were then calculated for each student in the sample.

##### Habits of Mind

The habits of mind variable was indicated by nine items from the DLE survey, and measured characteristics associated with academic success, which are seen as the foundation for lifelong learning (CIRP Construct Report, 2008). Students were asked, “How often in the past did you” and rated the frequency of each of the following: *support your opinions with a logical argument*, *seek solutions to problems and explain them to others*, *evaluate the quality or reliability of information you received*, *take a risk because you felt you had more to gain*, *seek alternative solutions to problems*, *look up scientific research articles and resources*, *explore topics on your own even though it was not required for a class*, *accept mistakes as part of the learning process*, and *analyze multiple sources of information before coming to a conclusion*. Responses were rated on a three-point scale: 1—“Not at all,” 2—“Occasionally,” and 3—“Frequently” for each item. The variables included in the model did not pass the MCAR test (χ^2^ = 82.919, df = 48, p = 0.01) (Little, 1988), and therefore listwise deletion was used as opposed to FIML to handle missing data. Five hundred and seventy cases out of the 580 cases in the sample were used in this model. The unidimensional confirmatory model evidenced adequate fit (χ^2^ = 144.445, p < 0.01, df = 27, CFI = 0.970, RMSEA = 0.089, SRMR = 0.052), and Cronbach’s alpha also indicated adequate internal consistency of the items (α = 0.86). Lastly, *habits of mind* factor scores based on this confirmatory model were calculated for each student in the sample.

Factor scores of the aforementioned predictor variables for autistic and non-autistic groups are summarized in Table 3. Additionally, factor loadings for all five latent variables and reliability coefficients are summarized in Table 4.

**Table 3:** Summary of descriptive statistics for predictor variables

	Autistic sample (N = 290) *M(SD)*	Non-autistic sample (N = 290) *M(SD)*	p
School involvement factor score	3.99 (0.72)	4.01 (0.63)	> 0.05
Faculty support factor score	12.10 (2.20)	12.11 (2.11)	> 0.05
Habits of mind factor score	16.64 (2.86)	16.58 (2.74)	> 0.05

**Table 4:** Factor loadings and reliability coefficients for latent variables

Latent variable	Indicator variables	Loading	Cronbach’s α
Discrimination	Verbal comments	0.77	0.83
	Cyberbullying	0.785	
	Exclusion	0.736	
	Offensive visual images	0.74	
	Anonymous phone calls	0.521	
Harassment	Physical assaults or injuries	0.913	0.85
	Threats of physical violence	0.87	
	Damage to personal property	0.696	
	Been sexually harassed	0.606	
School involvement	Joined a social fraternity or sorority	0.77	0.9
	Participated in leadership training	0.64	
	Joined an ethnic or culturally based fraternity or sorority	0.962	
	Participated in a student-run political club	0.559	
	Joined a racial/ethnic student organization	0.668	
Faculty support	Faculty encouraged me to ask questions and participate in discussion	0.509	0.83
	Faculty empower me to learn here	0.711	
	Faculty believe in my potential to succeed academically	0.899	
	At least one staff member has taken an interest in my development	0.692	
	At least one faculty member has taken an interest in my development	0.765	
Habits of mind	Support your opinions with a logical argument	0.781	0.86
	Seek solutions to problems and explain them to others	0.838	
	Evaluate the quality or reliability of information you received	0.784	
	Take a risk because you felt you had more to gain	0.594	
	Seek alternative solutions to problems	0.795	
	Look up scientific research articles and resources	0.616	
	Explore topics on your own even though it was not required for a class	0.698	
	Accept mistakes as part of the learning process	0.712	
	Analyze multiple sources of information before coming to a conclusion	0.856	

##### Co-occurring Developmental or Psychological Disabilities

During each survey year, students were asked to indicate whether they had learning disability, attention-deficit/hyperactivity disorder (ADHD), and/or psychological disorder (e.g., depression, anxiety, PTSD) by selecting all that apply to the following question: “Do you have any of the following disabilities or medical conditions?” Response options for each item included 1—“No,” and 2—“Yes”.

### Analyses

#### Discrimination and Harassment Experiences

To test for group differences between autistic college students and their non-autistic peers on their experiences of discrimination and harassment during their college years, two independent sample t-tests were conducted with the α level of 0.05. To confirm the statistical significance, bootstrapping was performed with the number of samples set at 1000. All analyses were conducted using SPSS version 27.

#### Predictors of Discrimination and Harassment

To investigate factors associated with college students’ discrimination and harassment experiences, multiple regression was conducted with the entire sample (N = 580). *Discrimination* and *Harassment* served as the outcome variables for each model, and the following variables entered each model in the following order hierarchically in two blocks: (1) diagnoses (i.e., learning disability, ADHD, psychological disorder, autism spectrum disorder), and (2) predicting factors (i.e., Faculty Support factor score, School Involvement factor score, Habits of Mind factor score) and interaction terms (i.e., ASD* Faculty Support factor score, ASD* School Involvement factor score, ASD* Habits of Mind factor score). The order of the entry was decided based on the assumption that co-occurring diagnoses may exert influence on the effects of predicting factors.

Assumptions for multiple regression were tested for the *discrimination* model and the *harassment* model with the predictor variables that entered each model. To test the assumption of linear relationship between the IVs and the DV, scatter plots were generated for both the *discrimination* model and the *harassment* model. Upon visual analysis, no violation of assumption was detected. In order to test the assumption that there is no multicollinearity in the data, analysis of collinearity statistics was conducted. VIF scores were well below 10, ranging between 1.001 and 1.130 for the *discrimination* model and 1.002 and 1.128 for the *harassment* model. Further, the tolerance scores were all above 0.2, ranging from 0.885 to 0.999 for the *discrimination* model and 0.887 to 0.998 for the *harassment* model. To test the assumption of independent residuals, the Durbin-Watson statistic was conducted. The Durbin-Watson statistic was 2.058 for the *discrimination* model and 1.960 for the *harassment* model. To test the assumption that the residuals are normally distributed, the P-P plot for the model was generated. The P-P plot suggested that the assumption of normality had been met. Lastly, to test the assumption that there are no influential cases biasing the final model, Cook’s Distance was calculated. Cook’s Distance values were all under 1 for both models, suggesting individual cases were not unduly influencing the model. In summary, no violations to these assumptions were found in either model. The above analyses were conducted using SPSS version 27.

## Results

### Demographic Characteristics

Descriptive characteristics of the sample are summarized in Table 5. The majority of students in the matched sample were White (63.4%) and attended 4-year colleges (62.1%). Only a small portion of the sample (11.4%) were first-generation college students.

**Table 5:** Demographic information of participants

Student characteristic N (%)	ASD (N = 290)	NT (N = 290)
Race		
Whilte	184 (63.4%)	184 (63.4%)
Two or more races/non-Hispanic	41 (14.1%)	34 (11.7%)
Hispanic—any race	19 (6.6%)	42 (14.5%)
Asian/American Indian/Hawaiian	31 (10.7%)	19 (6.6%)
Black/non-Hispanic	9 (3.1%)	11 (3.8%)
Unknown	6 (2.1%)	0 (0.0%)
First generation college students	33 (11.4%)	33 (11.4%)
Gender identity		
Male	136 (46.9%)	136 (46.9%)
Female	127 (43.8%)	127 (43.8%)
Other	27 (9.3%)	27 (9.3%)
Annual family income		
under $30,000	98 (33.8%)	98 (33.8%)
$30,000–$50,000	34 (11.7%)	34 (11.7%)
$50,000–$100,000	77 (26.6%)	77 (26.6%)
$100,000–$200,000	65 (22.4%)	65 (22.4%)
$200,000 or more	16 (5.5%)	16 (5.5%)
Institution type		
University	50 (17.2%)	50 (17.2%)
4 Year College	180 (62.1%)	180 (62.1%)
2 Year College	60 (20.7%)	60 (20.7%)

### Discrimination

Autistic and non-autistic students did not differ in their perceived experiences of discrimination with *t*(572) = − 1.049, *p* > *0.05*, *d* = − 0.088 (95% CI: − 0.251, 0.076).

### Harassment

Autistic and non-autistic students did not differ in their perceived experiences in harassment with *t*(568) = − 1.264, p > 0.05, *d* = − 0.106 (95% CI: − 0.270, 0.058).

### Factors Associated with Discrimination and Harassment Experiences

#### Discrimination

Full model results for all covariates for *discrimination* are summarized in Table 6. The results from the multiple regression analysis revealed a significant regression equation (F(10, 527) = 20.762, p < 0.001), with an ${R}^{2}$ of 0.283. Controlling for learning disability, ADHD, and autism statuses, having a psychological disorder significantly predicted more reported discrimination (*β* = 0.099, *p* = 0.010, ${F}^{2}=0.01$; small effect). Further, controlling for all disability statuses, students’ *school involvement* also significantly predicted greater reported discrimination (*β* = 0.330, *p* < 0.001, ${F}^{2}=0.05$; small effect), however *faculty support* and *habits of mind* significantly predicted less reported discrimination (*β* = − 0.125, *p* = 0.028, ${F}^{2}=0.01$; small effect) and (*β* = -0.125, *p* = 0.028, ${F}^{2}=0.01$; small effect) respectively. In addition, there was a significant positive interaction between autism status and *school involvement* (*β* = 0.632, *p* = 0.007, ${F}^{2}=0.01$; small effect).

**Table 6:** Multiple regression model summary for Discrimination

Outcome	Predictors	Standardized *β*	S.E	t-ratio	p	${F}^{2}$
Discrimination	Disability: Learning disability (dyslexia, etc.)	0.072	0.250	1.883	0.060	< 0.01
	Disability: Attention-deficit/hyperactivity disorder (ADHD)	0.067	0.230	1.741	0.082	< 0.01
	Disability: Psychological disorder (depression, anxiety, PTSD, etc.)	0.099*	0.217	2.593	0.010*	0.01
	Disability: Autism spectrum disorder	− 0.028	1.872	− 0.082	0.934	< 0.01
	Faculty support	− 0.125*	0.074	− 2.199	0.028*	0.01
	School involvement	0.330*	0.238	5.775	< .001*	0.05
	Habits of Mind (HoM)	− 0.125*	0.057	2.202	0.028*	0.01
	Autism* faculty support	− 0.151	0.101	− 0.665	0.506	0.01
	Autism* school involvement	0.632*	0.316	2.720	0.007*	0.01
	Autism* HoM	− 0.421	0.078	− 1.762	0.079	< 0.01
Constant	− 1.848					
$R$-square	0.283					
F-ratio	20.762 (p < 0.001)					
n	538					

*p $$\le$$ 0.05

#### Harassment

Full model results for all covariates for *harassment* are summarized in Table 7. The results from the multiple regression analysis revealed a significant regression equation (F(10,525) = 28.273, p < 0.001), with an ${R}^{2}$ of 0.350. Controlling for ADHD, psychological disorders and autism statuses, having a learning disability significantly predicted greater reported harassment (*β* = 0.126, *p* < 0.001, ${F}^{2}=0.02$; small effect). In addition, controlling for learning disability, ADHD, and psychological disorders, having autism significantly predicted less reported harassment (*β* = -1.022, *p* = 0.001, ${F}^{2}=0.01$; small effect). Further, controlling for all disability statuses, students’ *school involvement* also significantly predicted greater reported harassment (*β* = 0.161, *p* = 0.003, ${F}^{2}=0.01$; small effect). However, controlling for all disability statuses *faculty support* significantly predicted lower reported harassment (*β* = -0.172, *p* = 0.002, ${F}^{2}=0.01$; small effect), and *school involvement* significantly predicted higher harassment (*β* = 0.161, *p* = 0.003, ${F}^{2}=0.01$; small effect). In addition, there was a significant positive interaction between autism status and *school involvement* (*β* = 1.541, *p* < 0.001, ${F}^{2}=0.06$; small effect), and a significant negative interaction between autism status and *habits of mind* (*β* =—0.594, *p* = 0.009, ${F}^{2}=0.01$; small effect).

**Table 7:** Multiple regression model summary for Harassment

Outcome	Predictors	Standardized *β*	S.E	t-ratio	p	${F}^{2}$
Harassment	Disability: Learning disability (dyslexia, etc.)	0.126*	0.159	3.472	< .001*	0.02
	Disability: Attention-deficit/hyperactivity disorder (ADHD)	0.072	0.147	1.957	0.051	< 0.01
	Disability: Psychological disorder (depression, anxiety, PTSD, etc.)	0.038	0.138	1.038	0.300	< 0.01
	Disability: Autism spectrum disorder	− 1.022*	1.189	1.437	0.001*	0.01
	Faculty support	− 0.172*	0.047	− 3.175	.002*	0.01
	School involvement	0.161*	0.151	2.966	.003*	0.01
	Habits of Mind (HoM)	0.032	0.036	0.588	0.557	< 0.01
	Autism* Faculty support	0.158	0.064	0.732	0.465	< 0.01
	Autism* School involvement	1.541*	0.200	63,963	< 0.001*	0.06
	Autism* HoM	− 0.594*	0.049	− 2.612	0.009*	0.01
Constant	5.988					
$R$-square	0.350					
F-ratio	28.273 (p < 0.001)					
n	536					

*p $$\le$$ 0.05

## Discussion

The current study compared a large sample of autistic and non-autistic college students matched on co-occurring diagnoses and demographic information on their reported discrimination and harassment experiences. Furthermore, we examined whether institutional-level factors (i.e., faculty support) and student-level factors (i.e., disability status, habits of mind, involvement in campus-facilitated activities) were associated with more discrimination and harassment experiences reported by autistic and non-autistic college students. Contrary to our hypothesis, the present study found that while holding other developmental and psychological disabilities constant, autistic students and non-autistic students did not differ on their reported discrimination and harassment on campus. Further, while students’ involvement in particular campus activities (e.g., fraternity or sorority events, political clubs, racial/ethnic organizations) was associated with increased vulnerability to these negative interpersonal experiences in general, having autism intensified such effect. However, extant institutional support (faculty support) was associated with fewer discrimination and harassment experiences for both autistic and non-autistic students. Further, student-level factors (habits of mind) were associated with fewer discrimination experiences for both autistic and non-autistic students. Lastly, while habits of mind was not associated with harassment experiences in general, particularly for autistic students, habits of mind was associated with fewer harassment experiences.

### Discrimination and Harassment among Autistic and Non-autistic Students

The results of the present study revealed an absence of effects of autism in autistic college students’ moderate to severe forms of negative interpersonal experiences. Such results are contrary to prior work that has demonstrated that autistic young adults report high levels of negative social and interpersonal experiences in college (Gelbar et al., 2015; Jackson et al., 2018; McLeod et al., 2019; Wainer et al., 2013; White et al., 2011). However, while previous studies investigated more subtle forms of negative social experiences (e.g., isolation, loneliness, difficulty making friends, feeling different, verbal bullying, being lied to) and mental health challenges, the current study examined more severe forms of peer victimization (e.g., offensive written comments, unwanted phone calls, physical or sexual assault, threats to physical violence and damage to personal property) in addition to the types of negative experiences explored previously.

Further, considering the high prevalence of co-occurring conditions such as learning disabilities, ADHD, and psychological disorders in autistic students that may impact their college experiences, it is important to control for these effects when examining the effect of autism. Accordingly, the current study found that learning disabilities and psychological disorders were associated with heightened risk for discrimination and harassment respectively. There is emerging evidence that co-occurring disabilities or psychological challenges play an important role in autistic college students’ overall experiences and adjustment (Baczewski et al., 2022; Sturm & Kasari, 2019). Therefore, there is an urgent need for future investigations on specific co-occurring diagnoses or mental health challenges that contribute to autistic students’ social and interpersonal experiences in college. It is also critical to note that attributing negative college experiences solely on autism without closer examination of the effects on co-occurring conditions and other demographic characteristics may not be appropriate.

Further, little is known about how such negative interpersonal interactions on campus influence college students’ overall school outcomes such as academic achievement, graduation rate, and sense of belonging on campus. It is critical that future studies explore the long-term school outcomes of discrimination and harassment on college students across various diagnostic and comorbidity groups.

### Involvement in Campus-Facilitated Events

Contrary to our hypothesis, the extent to which college students were involved in campus-facilitated events was found to be positively and modestly associated with experiencing higher rates of discrimination and harassment regardless of their diagnostic statuses, while such effects were moderately exacerbated for autistic students. Many of the activities measured by this construct are those that require extensive time commitment for students. As a result of this greater presence in social spaces and group events (e.g., political protests, scheduled social events), any students who participate in these activities may encounter a greater number of social experiences and exchanges, thus opening them up for a greater number of negative experiences with peers. However, as our finding indicates even higher risk for autistic students in such situations, it is imperative that researchers and institutions investigate the factors that may contribute to such difference.

In addition to experiencing heightened discrimination and harassment in certain social situations such as campus-facilitated events and activities, autistic college students may be experiencing added layers of challenges. While the current study did not examine adverse effects beyond discrimination and harassment, previous studies have found that autistic college students report less confidence in building social relationships when compared to their neurotypical peers, despite a clear desire and need for such relationships (Fernandes et al., 2021; Sturm & Kasari, 2019). Accordingly, autistic students report that they put forth extra effort to fit in and often force themselves to engage in social opportunities (Van Hees et al., 2015) and engage in masking at the expense of their mental health (Miller et al., 2021). There is emerging evidence that autistic people tend to engage in masking (i.e., suppression of natural characteristics or identities in order to “appear normal”) in certain social situations, which has negative impacts on autistic individuals’ mental health and increases the likelihood of burnout and suicidal ideation (Cassidy et al., 2018; Miller et al., 2021; Raymaker et al., 2020). Future work must examine if experiencing discrimination and harassment on campus in spite of their intentional effort to seek social opportunities negatively impacts their future engagement in school events. Additionally, effects of masking on autistic students’ perceived discrimination and harassment should be investigated.

Moreover, institution-level factors such as the diversity and inclusivity of the campus culture and openness of the student body are two major contributors to a student’s ability to feel socially integrated with their campus (Hurtado et al., 2015). One possible explanation for autistic students’ heightened discrimination and harassment experiences could be perceived stigma towards their autistic identity and a campus culture that lacks adequate support for neurodivergent and disabled people. Future research must investigate the extent to which autistic students perceive negative social experiences stemming from stigma around the disability.

With this said, interacting with peers and engaging in campus activities are essential to developing relationships with others and subsequent feelings of campus belonging. Interventions and campus-wide programming that promote inclusivity and a greater understanding of neurodiversity are urgently needed. In addition, research suggest that self-disclosure of autism diagnosis in social situations has positive impact on social acceptance (see Thompson-Hodgetts et al., 2020 for a review). It is essential that future work investigate various ways to decrease such negative experiences reported by autistic college students when they engage in campus-facilitated events on campus.

It is important to note that, in this study, students indicated their school involvement based on whether they participated in a social fraternity or sorority, leadership training, an ethnic or culturally based fraternity or sorority, a student-run political club, and/or a racial/ethnic student organization. These activities represent a set of social experiences that a student may have while in college but are not inclusive of the full range of social opportunities available on college campuses. Many of these school-affiliated groups are based on social identity (e.g., racial/ethnic or cultural background, political affiliation and beliefs). The saliency of social identity in these groups and activities is one possible explanation for greater reported experiences of harassment and discrimination on campus for autistic students. The school activities not measured in our school involvement construct include more hobby or interest-based groups or clubs (e.g., video game club, volunteer-based organizations). Future studies should explore autistic students’ experiences in different types of school activities, as well as their perceptions of discrimination within these spaces.

### Faculty Support

The present findings revealed few possible protective factors that may buffer against discrimination and harassment experiences for both autistic and non-autistic college students. First, we found that the more faculty support and validation that both autistic and non-autistic students report receiving during their college years, the less likely it was that they perceive discrimination and harassment towards themselves. This supports a recent finding that, the more supportive relationship students have with their faculty the more likely it is they feel a greater sense of belonging on campus, which mitigates the effects of discrimination and harassment (Hurtado et al., 2015).

However, emerging evidence demonstrates that faculty and staff in postsecondary institutions have limited knowledge and inaccurate information about autism and other developmental disabilities (Sniatecki et al., 2015; Tipton & Blacher, 2014). Faculty members often report feeling unprepared to effectively support autistic students (Zeedyk et al., 2019), and they report to have limited knowledge of autism and the resources available on campus (Sniatecki et al., 2015). Consequently, college students with disabilities report insufficient understanding of their disabilities and lower expectation from faculty (Hong, 2015), which may impact their perceived support. As such, there is a need for clear understanding of faculties’ knowledge and ability to support their autistic students, and for appropriate training to prepare them to foster supportive relationships.

### Habits of Mind

While college students’ habits of mind were found to be protective against reported experiences of discrimination for the entire sample, habits of mind were found to be particularly protective for autistic students against harassment, more severe forms of negative experiences.

As habits of mind are considered an important set of prerequisite skills for college readiness (Johnson, 2013; Sullivan, 2012), it is important that students with disabilities are equipped with these skills upon entering college, and that they have continued opportunities to develop these skills throughout their college years. Though no prior studies have examined the direct link between autistic college students’ habits of mind and their harassment experiences, there is evidence that these skills can be fostered in the first year of college. More specifically, high-impact practices can foster habits of mind through frequent faculty-student interactions, working on professor’s research projects, collaborating with peers on their studies, having discussions with peers regarding various topics such as course contents or about issues surrounding different race / ethnicity (Hurtado & DeAngelo, 2012). Engagement in high-impact practices is thus likely to be particularly necessary for autistic students, and opportunities to practice such skills should be provided from their early years in college. Further, college students’ habits of mind were found to be associated with the quality of their free time during their college years. More specifically, students’ habits of mind was positively associated with spending time socializing, engaging with clubs and planning for their future (Gebby, 2018). While there is more to be learned about the relationship between college students’ habits of mind and their reported negative social or interpersonal experiences, there appears to be links between these skills, faculty support, and social opportunities on campus. As findings from the current study indicate that habits of mind can be particularly protective against harassment for autistic students, further investigation is needed to parse out the underlying paths between habits of mind, faculty support, school involvement and harassment experiences among autistic college students in order to better understand such relationships and to make practical recommendations for methods of fostering positive experiences for autistic students on college campuses.

### Limitations & Future Directions

The data used in this study were self-reported. Although this can be considered a strength of the study as the findings reflect autistic students’ own perceptions, the constructs used in this study must not be interpreted as an objective measure due to the nature of self-reported data. Future studies should go beyond measuring frequency of reported discrimination and harassment experiences to explore these incidents in more depth. Further, due to the written nature of survey questions, some questions can be interpreted subjectively. For example, it is possible that ‘family income’ be misinterpreted as the students’ own income, as some students may have their earnings. Additionally, experiencing “offensive visual images or items” can be due to unintentional exposure or offenders’ deliberate intent to engage in discrimination. Interview studies may be particularly well-suited for in-depth analysis of lived experiences of autistic students in regard to discrimination and harassment in college. Studies that partner with autistic students to conduct this research are necessary in order to understand the priorities and perspectives of autistic young adults.

Additionally, while there is a possibility of some students attending more than one institution, such information is not captured in the current analysis. Further, while gender fluidity is prevalent in the autistic population (Hillier et al., 2020), gender diversity is not captured in the current analysis due to less than 10% (N = 27) of the autistic group identifying as other than ‘male’ or ‘female.’ Future studies must disaggregate gender diversity within the autistic population and examine how their gender identity or sexual orientation influences their reported discrimination or harassment experiences.

Moreover, it is noteworthy that the majority of the autistic students in our sample are White and attend 4-year colleges. Given that the HERI survey is intended to be measure the experiences of a representative sample of college students in the U.S., these data may be more reflective of autistic student characteristics who are given the opportunity to go to 4-year college. Improvement in K-12 inclusive education, and encouragement of all students to attend college may broaden representation in future waves of the survey. Future studies with more diverse samples of autistic college students are needed in order to accurately portray their interpersonal experiences on campus.

### Implications and Recommendations

The current findings direct us to look beyond autistic traits of college students to acknowledge the context and environmental factors that may impact their challenges during college years. As faculty support was found to be beneficial for students across diagnostic groups and non-autistic students, institution-wide efforts to scaffold connections between faculty and students may be beneficial in fostering positive college experiences. Specifically, high-impact practices (HIP)—higher-order learning processes that involve problem solving, critical thinking and analytical skills to promote active learning and engagement—have been linked to increased academic success, sense of belonging, and retention (Kuh, 2008). HIPs have been found to be particularly beneficial for those students with minoritized identities (Sweat et al., 2013). Some possible ways to foster close faculty-student connection through high-impact practices may include creating close-knit learning communities and strengthening departmental support of students within each school. As faculty support and habits of mind are found to be related (Hurtado & DeAngelo, 2012), it is important that students receive early, pre-college exposure to habits of mind and professors examine ways to integrate skills related to habits of mind into their curriculum. Transition programs prior to and upon entering college that fosters skills related to college-readiness and habits of mind can support autistic students’ college experiences (White et al., 2021). Additionally, providing institution-wide opportunities to start building relationships with faculty, staff, and peers, as well as to familiarize students with available campus-facilitated activities prior to entering college through various modalities (e.g., orientations, school visits, summer camps, online meetings) can be especially beneficial for autistic students. While enrolled in postsecondary education, students can build important skills relating to habits of mind (e.g., flexible and innovative thinking, use of evidence to evaluate problems) through engagement with high-impact practices in their courses and through interactions with faculty and faculty research. Therefore, the faculty’s encouragement of involvement through these high-impact practices may be particularly helpful for autistic students to have positive experiences during their college years. Training faculty members to more effectively support students with disabilities is another method to enhance faculty support. Many faculty members express their desire for more training on ways to effectively support students with disabilities (Sniatecki et al., 2015; Zeedyk et al., 2019), and validated training curriculums specifically targeted for faculty to support autistic students in higher education are available (Debrand & Salzberg, 2005; Waisman et al., 2022).

Moreover, it is important for faculty and the institution to understand how autistic college students perceive faculty and institutional support. Some autistic students avoid disclosing their disability and seeking faculty support due to the possibility of lowered expectations, stigma, judgement, or being treated differently than other students (Hong, 2015). As autism is often considered an “invisible disability,” students can either use masking strategies or avoid seeking accommodations or support, which can in turn hinder successful integration to their college community. Once support programs are in place, it is important that the institutions seek autistic students’ feedback during and after the receipt of available supports to allow programmatic tailoring to a student's specific needs. Taken together, it is important that faculty and staff play proactive roles in promotion and outreach for available support, and delivery of the support should be welcoming but discreet.

Colleges should also foster an inclusive culture that embrace inclusivity and diversity, where neurodiversity is seen as a valuable asset to the campus community. Positive attitudes towards people with disabilities can be fostered through (1) sufficient information about disability and (2) frequent contact with persons with a disability (Gillespie-Lynch et al., 2015; Huskin et al., 2018; Zeedyk et al., 2019). A campus-wide effort to increase inclusivity and understanding of neurodiversity is one way to decrease discrimination and harassment. Systematic changes that increase acceptance of individual differences allow a campus to become a safer place for autistic students to engage in student events without fear of harassment. Greater education of both faculty and the student body at large may decrease the likelihood of students with disabilities of encountering negative experiences.

Finally, when autistic youth and their families make college choices, it is advised that they investigate the quality of faculty-student contact, such as teacher-to-student ratio, the student-faculty relationship, and opportunities for one-to-one or small group contacts. In addition, the state of individualized support for autistic students and neurodiversity awareness of the student body and faculty may be important considerations. Utilizing existing resources and looking into schools’ practices and effort such as campus-wide training for neurodiversity, campus culture that celebrates individuals’ unique strengths, and the level of inclusivity of school-facilitated activities and events is likely to set autistic students up for successful college experiences.
